# Polypharmacy, comorbidity and frailty: a complex interplay in older patients at the emergency department

**DOI:** 10.1007/s41999-022-00664-y

**Published:** 2022-06-20

**Authors:** Carmen S. van Dam, Helena A. Labuschagne, Kris van Keulen, Cornelis Kramers, Emma E. Kleipool, Emiel O. Hoogendijk, Wilma Knol, Prabath W. B. Nanayakkara, Majon Muller, Marijke C. Trappenburg, Mike J. L. Peters

**Affiliations:** 1 Department of Internal Medicine and Geriatrics, Amsterdam UMC, Location VUmc, Amsterdam, the Netherlands; 2 Department of Pharmacy, Amstelland Hospital, Amstelveen, the Netherlands; 3 Department of Pharmacology-Toxicology, Radboud University Medical Center, Nijmegen, the Netherlands; 4 Department of Epidemiology and Data Science, Amsterdam UMC, Location VUmc, Amsterdam, the Netherlands; 5 Department of Geriatric Medicine, University Medical Center Utrecht, Utrecht University, Utrecht, the Netherlands; 6 Section General and Acute Internal Medicine, Amsterdam Public Health Research Institute, Amsterdam UMC, Location VUmc, Amsterdam, the Netherlands

**Keywords:** Polypharmacy, Comorbidity, Frailty, Emergency department, Older adults

## Abstract

**Purpose:**

Older adults at the emergency department (ED) with polypharmacy, comorbidity, and frailty are at risk of adverse health outcomes. We investigated the association of polypharmacy with adverse health outcomes, in relation to comorbidity and frailty.

**Methods:**

This is a prospective cohort study in ED patients  ≥ 70 years. Non-polypharmacy was defined as 0–4 medications, polypharmacy 5–9 and excessive polypharmacy ≥ 10. Comorbidity was classified by the Charlson comorbidity index (CCI). Frailty was defined by the Identification of Seniors At Risk—Hospitalized Patients (ISAR-HP) score. The primary outcome was 3-month mortality. Secondary outcomes were readmission to an ED/hospital ward and a self-reported fall < 3 months. The association between polypharmacy, comorbidity and frailty was analyzed by logistic regression.

**Results:**

881 patients were included. 43% had polypharmacy and 18% had excessive polypharmacy. After 3 months, 9% died, 30% were readmitted, and 21% reported a fall. Compared with non-polypharmacy, the odds ratio (OR) for mortality ranged from 2.62 (95% CI 1.39–4.93) in patients with polypharmacy to 3.92 (95% CI 1.95–7.90) in excessive polypharmacy. The OR weakened after adjustment for comorbidity: 1.80 (95% CI 0.92–3.52) and 2.32 (95% CI 1.10–4.90). After adjusting for frailty, the OR weakened to 2.10 (95% CI 1.10–4.00) and OR 2.40 (95% CI 1.15–5.02). No significant association was found for readmission or self-reported fall.

**Conclusions:**

Polypharmacy is common in older patients at the ED. Polypharmacy, and especially excessive polypharmacy, is associated with an increased risk of mortality. The observed association is complex given the confounding effect of comorbidity and frailty.

**Supplementary Information:**

The online version contains supplementary material available at 10.1007/s41999-022-00664-y.

## Key summary points

**Aim:**

To investigate the association of polypharmacy with adverse health outcomes, in relation to comorbidity and frailty.

**Findings:**

Excessive polypharmacy (≥ 10 medications) is highly prevalent in older adults at the emergency department and associated with falls, mortality and readmission. Frailty and comorbidity partly drive the association of polypharmacy with adverse health outcomes.

**Message:**

Trials that target polypharmacy and inappropriate prescribing are needed to answer the lingering question of causality in the observed polypharmacy–mortality association and to evaluate whether medication review improves health outcomes in older patients at the ED.

**Supplementary Information:**

The online version contains supplementary material available at 10.1007/s41999-022-00664-y.

## Introduction

The proportion of older patients seen at the emergency department (ED) is high and is expected to rise in the next decades [1]. Since the number of medical problems increases with age, the number of pharmacological interventions increases as well. Thirty to forty-five percent of the Dutch older population receives five or more different medications (i.e., polypharmacy) and almost 20% receives ten or more medications (i.e., excessive polypharmacy) [2, 3].

Pharmacological therapy is a highly valued and an effective intervention. Nonetheless, the benefits of treatment should outweigh the risks in each individual patient, particularly in older patients with frailty, chronic comorbidity, or those near the end of life. The presence of multiple diseases, and therewith polypharmacy, increases the risk of drug non-compliance, drug–drug interactions, and adverse drug reactions (including readmission, falls and mortality) [4, 5]. Older patients with a higher degree of frailty may be more likely to experience adverse drug events, because of their reduced functional reserve and impaired homeostatic compensatory mechanisms [6]. Conversely, polypharmacy might contribute to frailty [7].

Studies investigating the association between polypharmacy and adverse events in older patients in the emergency care setting are sparse, and did not include important confounders such comorbidity and frailty [8–13]. The aim of this study is to assess the prevalence of (excessive) polypharmacy in older patients at the ED. Second, we investigate the association between polypharmacy and adverse health outcomes, and the extent to which chronic comorbidity and frailty account for this association.

## Methods

### Study design and setting

This prospective cohort study—the Amsterdam Geriatric Emergency Medicine study (AmsterGEM) [14]—was conducted at the ED of two Dutch hospitals: tertiary academic hospital Amsterdam UMC location VUmc in Amsterdam and the general community hospital Amstelland in Amstelveen. Data were collected on a daily basis from November 2017 to June 2018, mostly during office hours, and during a limited number of evenings and weekend days. All participants or their legally authorized representative provided written informed consent. This study was approved by the medical ethical board of Amsterdam UMC, location VUmc.

### Participants

For the AmsterGEM study [14], research students screened every patient aged ≥ 70 years attending the ED for eligibility, regardless of the reason for presentation and/or specialty they presented for. Exclusion criteria were patients labeled as high urgency (according to the Manchester Triage System—code red [15]), language barrier, unknown number of prescriptions, limited length of stay at the ED, or inability to give informed consent (for example due to altered mental status in the absence of a caregiver who could provide informed consent by proxy). Written informed consent was obtained from all participants or their caregivers by proxy.  This study was performed in line with the principles of the Declaration of Helsinki. Approval was granted by the Ethics Committee of Amsterdam University Medical Center.

### Data collection

Data were collected by chart review and interviews with patients and their caregivers at the ED. All the research students collecting data were extensively trained by a team of geriatric consultants. Sociodemographic data and care-related data were obtained at baseline, including living situation, and number of prescriptions. Physical status was assessed by ‘The Katz Index of Independence in Activities of Daily Living’ (Katz-ADL [16]: ranging from 0–6, with a score of 0 indicating independence). The Charlson comorbidity index (CCI) was used to classify chronic comorbidity [17]. Frailty was defined by the Identification of Seniors At Risk—Hospitalized Patients (ISAR-HP) score to obtain their frailty status. The ISAR-HP is a validated frailty screening instrument, which was developed in a cohort study of hospitalized patients in the Netherlands. The ISAR-HP was chosen because of it is a frequently used and internationally acknowledged screening instrument, and its prognostic accuracy is comparable with other screening instruments [18–21]. A score of ≥ 2 is the cutoff value for a positive score, indicating an increased risk of adverse health outcomes (frail) [18]. In line with the original studies of the instruments (Katz and ISAR-HP), we asked how a patient functioned physically and cognitively two weeks prior to the ED visit to rule out interference of the acute illness.

### Polypharmacy

Patients were asked how many medications they used, and this was verified with the Electronic Health Record. If the patient was not able to answer this question, the accompanying caregiver was asked. Vitamins, supplements and topical drugs were excluded, except for thiamine and vitamin D. The number of medications was documented, and classified as non-polypharmacy (0–4), polypharmacy (5–9) and excessive polypharmacy (≥ 10) [2, 22]. Substances in combination tablets were calculated separately. Both long-term and short-term prescriptions (e.g., antibiotics or incidental painkillers) were included. The pharmacological type of medication was not further specified.

### Outcome measures and follow-up

The primary outcome measures was 3-month mortality. Secondary outcome measures were readmission and a self-reported fall at 3 months. Other follow-up timepoints were at 1 and 6 months. Follow-up data were collected by research students, who were not blinded to baseline data. Follow-up information was obtained by telephone using a standardized charting form. If the patient was unreachable after five attempts by telephone, follow-up data were obtained from the general practitioner. Data on mortality were extracted from the electronic health record and cross-referenced with the general practitioner or caregiver. A fall was defined as an event reported by the person who fell. At each follow-up moment, we asked the patient ‘*did you fall between now and inclusion*?’ by telephone and registered a fall as a dichotomous answer (yes/no). If possible, the caregiver was asked to verify the answer. Only the first fall after baseline was taken into consideration. Readmission was defined as a second presentation at the ED and/or readmission to a hospital ward.

### Statistical analysis

Continuous variables were displayed as median with an interquartile range (IQR) given the skewed data. Dichotomous variables were presented as numbers with percentages. Differences between groups were tested with a Mann–Whitney *U* test. Differences in dichotomous data were evaluated with a Chi-square test. In all analyses, a *p*-value of < 0.05 was considered statistically significant. Logistic regression analyses were performed to compare the risk of mortality, readmission, and a self-reported fall in patients with polypharmacy, and excessive polypharmacy, with non-polypharmacy as reference category. Different models were conducted: (1) crude; (2) adjusted for age and gender; (3) adjusted for chronic comorbidity (Charlson comorbidity index) and (4) adjusted for frailty (ISAR-HP). Before conducting the logistic regression analyses, we checked for collinearity among polypharmacy, comorbidity and frailty, using Spearman correlation coefficients and variance inflation factors (VIFs). In our data, there was no substantial overlap between these variables (Spearman correlation ranged from 0.28 to 0.42 and all VIFs were < 2). Additional logistic regression analyses were conducted to evaluate the association between the use of one additional medication and adverse outcomes in all patients, and expressed as odd ratio’s with 95% confidence intervals. A sensitivity analysis was performed for the primary outcome of falls after 3 months in patients without self-reported memory problems. Data were statistically analyzed with IBM SPSS statistics version 25 (IBM Corp., Armonk, N.Y., USA).

## Results

### Patients

**Fig. 1:**
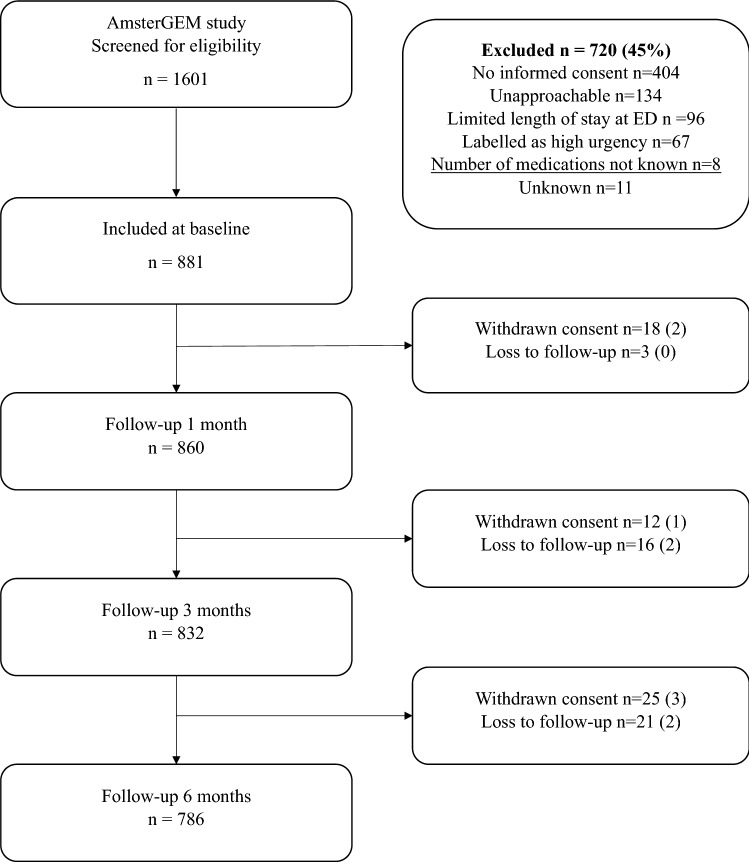
Flowchart study population. Numbers displayed as *n* (%)

In total, 1601 patients were screened for eligibility and 720 were excluded (Fig. 1). Most patients had to be excluded because no informed consent could be given (often because a caregiver was absent to provide consent for a patient who was too ill or confused). The number of patients with informed consent by proxy was not noted. 134 patients were considered unapproachable according to the medical staff at the ED (e.g., patients that just received bad news or patients in extreme pain). 96 patients were excluded due to their limited length of stay at the ED (e.g., patients that were admitted to a hospital ward or transferred to a different hospital before the research student could approach them). No reason of exclusion was reported in 11 patients. For this study, eight patients were excluded due to missing data on number of medications. In total, 881 patients were included, of whom 832 patients (94%) had data available at 3-month follow-up.

### Baseline characteristics

**Fig. 2:**
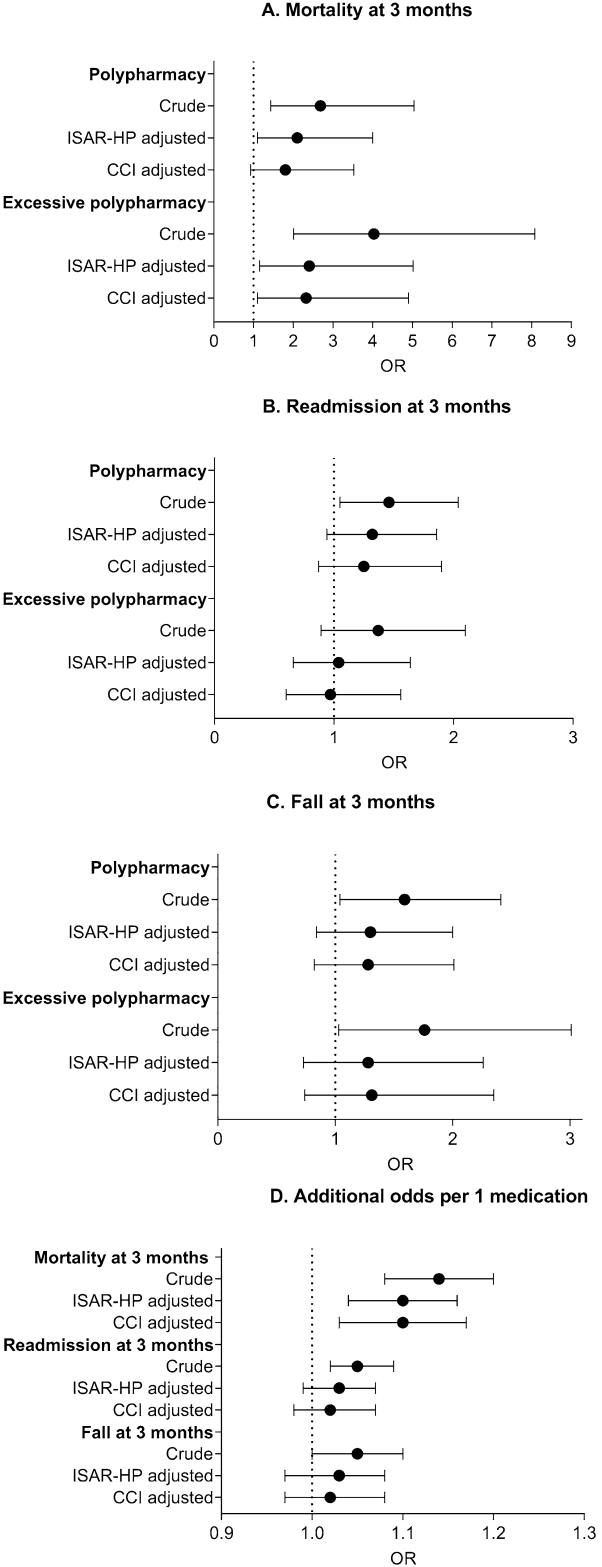
Associations between polypharmacy and adverse outcomes at 3 months for mortality (panel **A**), readmission (panel **B**), fall (panel **C**) and additional odds per 1 medication (panel **D**). *ISAR-HP* identification of seniors at risk—hospitalized patients; *CCI* Charlson comorbidity index score

Table 1 presents an overview of the baseline characteristics of the study population. Of all patients, 380 (43%) and 159 (18%) patients matched the criteria of polypharmacy and excessive polypharmacy respectively. Patients with polypharmacy and excessive polypharmacy were more often male (48% and 56%, compared with 43%, respectively), living in a nursing home and reported more cognitive complaints as compared with non-polypharmacy patients. Polypharmacy patients had a higher burden of disease according to the Charlson comorbidity index (median 5 (polypharmacy) and 6 (excessive polypharmacy), compared with 4 for non-polypharmacy), and a higher degree of frailty according to the ISAR-HP score (Fig. 2).

**Table 1:** Baseline characteristics

	Total population *N* = 881	Non-polypharmacy *N* = 342 (39%)	Polypharmacy *N* = 380 (43%)	Excessive polypharmacy *N* = 159 (18%)	*p*-value
Age, median [IQR]	78 [74–85]	77 [73–84]	79 [74–85]	78 [73–84]	0.028*
Male, *N* (%)	417 (47)	146 (43)	182 (48)	89 (56)	0.006*
Education after 14 years of age, *N* (%)	671 (76)	267 (78)	287 (76)	117 (74)	0.247
Living situation, *N* (%)					0.000*
Home without home help	460 (52)	208 (61)	185 (49)	67 (42)	
Home with home help	362 (41)	122 (36)	168 (44)	72 (45)	
Institute	59 (7)	12 (3)	27 (7)	20 (13)	
Self-reported memory problems, *N* (%)	189 (22)	47 (14)	95 (25)	47 (30)	0.000*
Katz score, median [IQR]	0 [0–1]	0 [0–0]	0 [0–1]	0 [0–2]	0.000*
Fall previous 6 months, *N* (%)	400 (45)	147 (43)	184 (48)	69 (43)	0.635*
ISAR score					0.000*
0	275 (31)	148 (43)	98 (26)	29 (18)	
1	171 (19)	72 (21)	73 (19)	26 (16)	
2	91 (10)	37 (11)	42 (11)	12 (7)	
3	134 (15)	37 (11)	70 (18)	27 (17)	
4	164 (19)	39 (11)	76 (20)	49 (31)	
5	46 (5)	9 (3)	21 (6)	16 (11)	
ISAR-HP ≥ 2, *N* (%)	435 (49)	122 (36)	209 (55)	104 (65)	0.000*
CCI, median [IQR]	5 [4–6]	4 [3–5]	5 [4–7]	6 [5–8]	0.000*
Presenting complaint at ED					0.646
Fall	203 (23)	96 (28)	85 (22)	22 (14)	
Non-specific complaint	83 (9)	18 (5)	44 (12)	21 (13)	
Cardiopulmonary disease	258 (29)	88 (26)	113 (30)	57 (36)	
Gastro-intestinal disease	87 (10)	27 (8)	46 (12)	14 (9)	
Neurological disease	66 (8)	31 (9)	23 (6)	12 (12)	
Dermatological disease	41 (5)	19 (6)	10 (3)	12 (12)	
Infectious disease	35 (4)	12 (4)	16 (4)	7 (4)	
Musculoskeletal disease	35 (4)	17 (5)	15 (4)	3 (2)	
Nephrogenic/urogenital disease	27 (3)	10 (3)	10 (3)	7 (4)	
Trauma other than fall	17 (2)	14 (4)	2 (1)	1 (1)	
Oncology related	11 (1)	5 (2)	5 (1)	1 (1)	
Deviation in blood results	9 (1)	4 (2)	5 (1)	0 (0)	
Miscellaneous	6 (1)	1 (0)	6 (2)	2 (1)	

*Non-polypharmacy* the use of 0–4 medications; *polypharmacy *the use of 5–9 medications; *excessive polypharmacy *the use of 10 or more medications; *ISAR-HP* identification of seniors at risk—hospitalized patients; *CCI *Charlson comorbidity index; *non-specific complaint* for example weakness or malaise without localized symptoms. Miscellaneous: for example allergic reaction, epistaxis, catheter problems

### Adverse health outcomes

After 3 months, a total 76 (9%) older patients had died, 249 (31%) patients were readmitted to the hospital, and 141 (21%) reported a fall (Table 2). In the next sections, we describe only the results for 3-month follow-up in the text. The results on 1- and 6-month follow-up outcomes are presented in Supplementary Tables 1 and 2.

**Table 2:** Prevalence of adverse outcomes

Adverse outcome	1 month *n* event/total *n*	%	3 months *n* event/total *n*	%	6 months *n* event/total *n*	%
Mortality	38/862	4	76/843	9	106/782	14
Readmission to ED or hospital ward	145/832	17	249/812	31	309/751	41
Fall	71/664	11	141/658	21	203/617	33

*Mortality* positive if patient had died between baseline and follow-up; *readmission *at least one readmission to ED or to hospital ward between baseline and follow-up; *fall* at least one fall between baseline and follow-up moment. The valid *N* varied between outcomes due to missing data on readmission and falls

### Mortality

The odds ratio (OR) for mortality ranged from 2.62 (95% CI 1.39–4.93) in patients with polypharmacy to 3.92 (95% CI 1.95–7.90) in patients with excessive polypharmacy, compared with non-polypharmacy (Table 3). This association weakened after adjustment for CCI: OR 1.80 (95% CI 0.92–3.52) for polypharmacy and OR 2.32 (95% CI 1.10–4.90) for excessive polypharmacy. After adjustment for ISAR-HP, the association weakened to OR 2.40 (95% CI 1.10–4.00) for polypharmacy and OR 2.40 (95% CI 1.15–5.02) for excessive polypharmacy (Fig. 2). For each additional medication, the OR for mortality increased by 14%, and by 7% after adjustment for chronic comorbidity or frailty (Fig. 2). The results for 1- and 6-month follow-ups are comparable.

**Table 3:** Association of polypharmacy with adverse outcomes at 3 months

	Prevalence *N* event/group (%)	Crude odds ratio (95% CI)	Adjusted odds ratio^†^ (95% CI)	Adjusted odds ratio* (95% CI)	Adjusted odds ratio^◊^ (95% CI)
Mortality 3 months					
Total population	76/832 (9)				
Non-polypharmacy	14/323 (4)	Reference	Reference	Reference	
Polypharmacy	39/360 (11)	2.68 (1.43–5.04)	2.62 (1.39–4.93)	2.10 (1.10–4.00)	1.80 (0.92–3.52)
Excessive polypharmacy	23/149 (15)	4.03 (2.01–8.08)	3.92 (1.95–7.90)	2.40 (1.15–5.02)	2.32 (1.10–4.90)
Additional odds per 1 medication		1.14 (1.08–1.20)	1.14 (1.08–1.20)	1.10 (1.04–1.16)	1.10 (1.03–1.17)
Readmission 3 months					
Total population	249/833 (30)				
Non-polypharmacy	83/335 (25)	Reference	Reference	Reference	
Polypharmacy	121/367 (33)	1.46 (1.05–2.04)	1.46 (1.04–2.04)	1.32 (0.94–1.86)	1.25 (0.87–1.90)
Excessive polypharmacy	47/148 (32)	1.37 (0.89–2.10)	1.30 (0.84–1.20)	1.04 (0.66–1.64)	0.97 (0.60–1.56)
Additional odds per 1 medication		1.05 (1.02–1.09)	1.05 (1.01–1.09)	1.03 (0.99–1.07)	1.02 (0.98–1.07)
Fall 3 months					
Total population	141/683 (21)				
Non-polypharmacy	45/285 (16)	Reference	Reference	Reference	Reference
Polypharmacy	68/300 (23)	1.59 (1.04–2.41)	1.45 (0.97–2.27)	1.30 (0.84–2.00)	1.28 (0.82–2.01)
Excessive polypharmacy	28/111 (25)	1.76 (1.03–3.01)	1.71 (0.99–2.93)	1.28 (0.73–2.26)	1.31 (0.74–2.35)
Additional odds per 1 medication		1.05 (1.00–1.10)	1.05 (1.00–1.10)	1.03 (0.97–1.08)	1.02 (0.97–1.08)

^**†**^ Adjusted for age, gender, * adjusted for age, gender, ISAR-HP score, ◊ adjusted for age, gender, CCI

### Readmission

The OR for readmission ranged from 1.46 (95% CI 1.04–2.04) in patients with polypharmacy to 1.37 (95% CI 0.89–2.10) in patients with excessive polypharmacy, compared with non-polypharmacy (Table 3). This association weakened after adjustment for CCI and ISAR-HP. For each additional medication, the OR for readmission increased with 5%, but also weakened after adjustment for chronic comorbidity and frailty (Fig. 2). The results for 1- and 6-month follow-ups are comparable.

### Fall

For self-reported falls, no association was found except for each additional medication (Table 3). This showed an increase of 5%, but this association also weakened after adjustment for chronic comorbidity and frailty (Fig. 2). The results for 1- and 6-month follow-up are comparable. A sensitivity analysis was performed for the primary outcome of falls after 3 months in patients without self-reported memory problems which showed the same results (data not shown).

## Discussion

This cohort study confirms that polypharmacy is highly prevalent in older patients at the ED. Polypharmacy was associated with increased 3-month mortality, and this association increased with the number of medications taken. We found no association between polypharmacy and readmission or a self-reported fall after adjustment for chronic comorbidity and frailty.

This study also illustrates that the observed polypharmacy–mortality association is complex given the confounding effect of chronic comorbidity and frailty. Strong associations between polypharmacy and adverse events have been frequently reported in older patients at the ED [11, 13]. However, studies evaluating this relationship while taking into account the possible confounding effect of chronic comorbidity and frailty are limited. In this study, older patients with polypharmacy have a roughly 2.5–4 times higher odds for mortality at 3 months compared to patients without polypharmacy. The odds ratio for mortality attenuated after adjustment for chronic comorbidity or frailty, but the odds ratio for mortality remained roughly twofold higher in patients with polypharmacy or excessive polypharmacy. We found no evidence for collinearity. These findings might suggest that medication use, frailty and comorbidity each act on adverse health outcomes through their own parallel pathophysiological mechanism.

It seems plausible that the onset of disease precedes the start of medication and results in adverse health outcomes, together with that progression of (multiple) diseases. With increasing frailty, medication related problems are more likely because of the reduced functional reserve and impaired homeostatic compensatory mechanisms [7, 23]. The risk–benefit ratio of a specific drug tends to increase in patients with comorbidity, and frailty. Thus polypharmacy may lead to harmful effects instead of beneficial effects [23]. This hypothesis is in line with a previous observation that polypharmacy is associated with an increased risk of adverse events in frail older patients, but not in non-frail older patients at the ED [9]. In line with previous literature, our study showed an increase in number of medication in line with an increase in frailty or comorbidity, suggesting a triangular relationship [7]. Comorbidity and frailty are often seen as the cause of polypharmacy, but the opposite might also be true [7, 23].

The high prevalence of polypharmacy, comorbidity, and frailty along with the high proportion of short-term adverse events in our cohort justify initiatives to improve the prescribing quality and a close medication review in older ED patients [24]. Results from the EQUiPPED [25] program using a multidisciplinary approach using a decision support tool showed a reduction in the proportion of potentially inappropriate medications prescribed to older patients discharged from the ED (< 5%) and a greater than 50% total reduction in the prescription of potentially inappropriate medications. In addition, two recently published feasibility studies demonstrated that a collaborative medication review and deprescribing intervention is feasible in older patients with polypharmacy at the ED [26, 27]. Despite this, there is still a lack of evidence to show that targeting polypharmacy in the ED improves patient outcomes. Yet, a close medication review seems reasonable near the end of life, to focus more on care instead of cure (for example, to reduce the number of preventive medications) [7, 23, 24]. Prescribers should be aware of co-occurrence of polypharmacy and frailty and cautious when prescribing new drugs.

The strengths and weaknesses of this study merit careful consideration. Major strengths are its prospective design, large sample size, and well-characterized study population. This is also the first study at the ED that evaluates frailty and comorbidity in relation to polypharmacy and adverse outcomes using the ISAR-HP [18] and Charlson comorbidity index [17].

Weaknesses should also be acknowledged. First, the possibility of selection bias, as older patients were only included if they were willing and/or able to participate. Second, inaccurate medication recall is common among older patients [28] and perhaps even more common in our cohort with a significant proportion of older patients with cognitive complaints (up to 30% in older patients with excessive polypharmacy). In this regard, it is important to note that we only included confused patients if a caregiver was present to provide informed consent by proxy, and if the caregiver was able to answer the questions on the physical and cognitive status of the patient two weeks prior to the ED visit. Recall bias also led to missing data on a self-reported fall with missing data in about 20% of the patients at 3 months. Third, information that might co-influence the risk of mortality is lacking, for example the acute illness severity at the ED, with potential underrepresentation of severe illness in this cohort. These potential sources of bias might lead to exclusion of a group of patients with a high risk of adverse events, and subsequently an underestimation of the relation of medication use with adverse outcomes. Since we excluded confused patients and patients triaged as ‘highly urgent’, severe illness might be underrepresented rather than overrepresented in this cohort. Last, we only assessed the number of medications taken at baseline which might have led to potential undetected changes in medication use during the course of follow-up. In addition, the exact type of medications and indications of use were not noted.

## Conclusion

This study in older patients at the ED shows that polypharmacy is highly prevalent and independently associated with mortality. However, this association was attenuated by frailty and comorbidity, illustrating a complex interplay. Trials that target polypharmacy and inappropriate prescribing are needed to address the unanswered question relating to causality in the observed polypharmacy–mortality association and to evaluate whether medication review improves health outcomes in older patients at the ED.

## Supplementary Information

Below is the link to the electronic supplementary material.

Supplementary file1 (DOCX 16 KB)

Supplementary file2 (DOCX 19 KB)
