# Supplementary material for: Polypharmacy, comorbidity and frailty: a complex interplay in older patients at the emergency department
Source: Eur Geriatr Med. 2022 Jun 20;13(4):849–57. doi: 10.1007/s41999-022-00664-y (PMC9378326; doi:10.1007/s41999-022-00664-y)
Supplement: Supplementary file 1 — Supplementary file1 (DOCX 16 KB) [file 41999_2022_664_MOESM1_ESM.docx]

**Supplementary Table 1. Association of polypharmacy with adverse outcomes at 1 month**

|  | **Prevalence**  **N event/group (%)** | **Crude odds ratio (95% CI)** | **Adjusted odds ratio† (95% CI)** | **Adjusted odds ratio* (95% CI)** | **Adjusted odds ratio◊ (95% CI)** |
| --- | --- | --- | --- | --- | --- |
| ***Mortality 1 month*** |  |  |  |  |  |
| Total population | 38/860 (4) |  |  |  |  |
| Non-polypharmacy | 7/337 (2) | Reference | Reference | Reference |  |
| Polypharmacy | 17/372 (5) | 2.26 (0.92 – 5.51) | 2.21 (0.90 – 5.42) | 1.56 (0.63 – 3.90) | 1.52 (0.58 – 3.98) |
| Excessive polypharmacy | 14/151 (9) | 4.82 (1.90 – 12.20) | 4.62 (1.82 – 11.74) | 2.18 (0.81 – 5.87) | 3.47 (1.27 – 9.48) |
| Additional odds per 1 medication |  | 1.14 (1.06 – 1.22) | 1.13 (1.06 – 1.22) | 1.07 (0.99 – 1.15) | 1.12 (1.03 – 1.21) |
| ***Readmission 1 month*** |  |  |  |  |  |
| Total population | 145/851 (17) |  |  |  |  |
| Non-polypharmacy | 48/326 (15) | Reference | Reference | Reference |  |
| Polypharmacy | 67/367 (18) | 1.34 (0.89 – 2.01) | 1.35 (0.89 – 2.02) | 1.28 (0.84 – 1.93) | 1.15 (0.75 – 1.77) |
| Excessive polypharmacy | 30/148 (20) | 1.53 (0.92 – 2.53) | 1.44 (0.86 – 2.39) | 1.28 (0.75 – 2. 18) | 1.07 (0.61 – 1.86) |
| Additional odds per 1 medication |  | 1.06 (1.02 – 1.11) | 1.06 (1.01 – 1.10) | 1.05 (1.00 – 1.10) | 1.03 (0.99 – 1.08) |
| ***Fall 1 month*** |  |  |  |  |  |
| Total population | 71/683 (10) |  |  |  |  |
| Non-polypharmacy | 27/295 (9) | Reference | Reference | Reference | Reference |
| Polypharmacy | 36/295 (12) | 1.30 (0.77 – 2.21) | 1.21 (0.71 – 2.06) | 1.00 (0.58 – 1.73) | 0.97 (0.55 – 1.71) |
| Excessive polypharmacy | 8/108 (7) | 0.75 (0.33 – 1.71) | 0.72 (0.31 – 1.64) | 0.48 (0.20 – 1.14) | 0.49 (0.21 – 1.18) |
| Additional odds per 1 medication |  | 1.01 (0.95 – 1.07) | 1.00 (0.94 – 1.07) | 0.97 (0.90 – 1.04) | 0.97 (0.90 – 1.04) |

***†*** *adjusted for age, gender,* ******* *adjusted for age, gender, ISAR-HP score, ◊ adjusted for age, gender, CCI*
