# Supplementary material for: Polypharmacy, comorbidity and frailty: a complex interplay in older patients at the emergency department
Source: Eur Geriatr Med. 2022 Jun 20;13(4):849–57. doi: 10.1007/s41999-022-00664-y (PMC9378326; doi:10.1007/s41999-022-00664-y)
Supplement: Supplementary file 2 — Supplementary file2 (DOCX 19 KB) [file 41999_2022_664_MOESM2_ESM.docx]

**Supplementary Table 2. Association of polypharmacy with adverse outcomes at 6 months**

|  | **Prevalence**  **N event / group (%)** | **Crude odds ratio (95% CI)** | **Adjusted odds ratio† (95% CI)** | **Adjusted odds ratio* (95% CI)** | **Adjusted odds ratio◊ (95% CI)** |  |
| --- | --- | --- | --- | --- | --- | --- |
| ***Mortality 6 months*** |  |  |  |  |  | |
| Total population | 106/786 (13) |  |  |  |  | |
| Non-polypharmacy | 24/303 (8) | Reference | Reference | Reference |  | |
| Polypharmacy | 52/337 (15) | 2.12 (1.27 – 3.54) | 2.08 (1.25 – 3.47) | 1.67 (0.99 – 2.82) | 1.41 (0.81 – 2.46) | |
| Excessive polypharmacy | 30/146 (4) | 3.01 (1.69 – 5.36) | 2.93 (1.64 – 5.24) | 1.80 (0.97 – 3.35) | 1.73 (0.92 – 3.27) | |
| Additional odds per 1 medication |  | 1.11 (1.06 – 1.16) | 1.11 (1.06 – 1.16) | 1.06 (1.01 – 1.12) | 1.08 (1.06 – 1.12) | |
| ***Readmission 6 months*** |  |  |  |  |  | |
| Total population | 309/775 (40) |  |  |  |  | |
| Non-polypharmacy | 106/335 (32) | Reference | Reference | Reference |  | |
| Polypharmacy | 158/367 (43) | 1.57 (1.14 – 2.17) | 1.58 (1.14 – 2.18) | 1.51 (1.08 – 2.10) | 1.41 (0.99 – 2.00) | |
| Excessive polypharmacy | 65/148 (44) | 1.58 (1.05 – 2.37) | 1.52 (1.01 – 2.29) | 1.39 (0.90 – 2.14) | 1.18 (0.75 – 1.86) | |
| Additional odds per 1 medication |  | 1.07 (1.03 – 1.10) | 1.06 (1.03 – 1.10) | 1.06 (1.02 – 1.10) | 1.04 (1.00 – 1.08) | |
| ***Fall 6 months*** |  |  |  |  |  | |
| Total population | 203/648 (31) |  |  |  |  | |
| Non-polypharmacy | 72/290 (25) | Reference | Reference | Reference | Reference | |
| Polypharmacy | 110/313 (35) | 1.52 (1.06 – 2.20) | 1.41 (0.97 – 2.06) | 1.29 (0.88 – 1.89) | 1.32 (0.89 – 1.97) | |
| Excessive polypharmacy | 36/113 (32) | 1.08 (0.66 – 1.78) | 1.01 (0.61 – 1.69) | 0.83 (0.49 – 1.41) | 0.88 (0.51 – 1.52) | |
| Additional odds per 1 medication |  | 1.02 (0.98 – 1.07) | 1.02 (0.97 – 1.06) | 1.00 (0.95 – 1.05) | 1.00 (0.96 – 1.05) | |

***†*** *adjusted for age, gender,* ******* *adjusted for age, gender, ISAR-HP score, ◊ adjusted for age, gender, CCI*
